# Decision-support systems for ambulatory care, including pandemic requirements: using mathematically optimized solutions

**DOI:** 10.1186/s12911-022-01866-x

**Published:** 2022-05-14

**Authors:** Neele Leithäuser, Dennis Adelhütte, Kristin Braun, Christina Büsing, Martin Comis, Timo Gersing, Sebastian Johann, Arie M. C. A. Koster, Sven O. Krumke, Frauke Liers, Eva Schmidt, Johanna Schneider, Manuel Streicher, Sebastian Tschuppik, Sophia Wrede

**Affiliations:** 1 Fraunhofer ITWM, Fraunhofer-Platz 1, 67663 Kaiserslautern, Germany; 2 FAU Erlangen, Cauerstraße 11, 91058 Erlangen, Germany; 3 RWTH Aachen University, Pontdriesch 10-12, 52062 Aachen, Germany; 4 TU Kaiserslautern, Gottlieb-Daimler-Straße 47, 67663 Kaiserslautern, Germany

**Keywords:** Decision support system, Mathematical optimization, Cartographic representation, Ambulatory care, Operations research

## Abstract

**Background:**

The healthcare sector poses many strategic, tactic and operational planning questions. Due to the historically grown structures, planning is often locally confined and much optimization potential is foregone.

**Methods:**

We implemented optimized decision-support systems for ambulatory care for four different real-world case studies that cover a variety of aspects in terms of planning scope and decision support tools. All are based on interactive cartographic representations and are being developed in cooperation with domain experts. The planning problems that we present are the problem of positioning centers for vaccination against Covid-19 (strategical) and emergency doctors (strategical/tactical), the out-of-hours pharmacy planning problem (tactical), and the route planning of patient transport services (operational). For each problem, we describe the planning question, give an overview of the mathematical model and present the implemented decision support application.

**Results:**

Mathematical optimization can be used to model and solve these planning problems. However, in order to convince decision-makers of an alternative solution structure, mathematical solutions must be comprehensible and tangible. Appealing and interactive decision-support tools can be used in practice to convince public health experts of the benefits of an alternative solution. The more strategic the problem and the less sensitive the data, the easier it is to put a tool into practice.

**Conclusions:**

Exploring solutions interactively is rarely supported in existing planning tools. However, in order to bring new innovative tools into productive use, many hurdles must be overcome.

## Background

Ambulatory medical care is an integral component of our healthcare system. This system currently faces major challenges to maintain the expected level of service: The current demographic change in the population of Germany prospectively leads to a greater need of medical healthcare services. Simultaneously, in rural areas, the population density is already low and will continue to decline in the future, so that the medical infrastructure in these regions cannot be operated economically anymore. In many cases, both aspects lead to centralization, e.g. in the form of medical centers in cities. The rural population therefore often has to accept long access distances (cf. [1]). At the same time, the public health sector is under increasing pressure due to limited resources and budgets. Additionally, the current pandemic situation poses further challenges due to the omnipresent risk of contagion: resource and personnel capacities are even more limited, virus protection methods permanently require time and money and the absence of personnel has to be taken into account.

Maintaining the healthcare services despite all these challenges as good as possible, leads to complex planning problems of various types.

Planners usually have a medical background and years of experience in their fields. However, in many medical domains, solutions to complex problems are still computed manually or with the the help of greedy strategies resulting in a rather local point of view. Hence, the full potential of mathematical optimization models to implement global-oriented and integrated decision-support systems is not exploited yet.

This work summarizes some of the results obtained by the authors while studying challenges for three main pillars of ambulatory care, namely for pharmacies, for emergency doctors, as well as for medical transport. Thereby, case studies emerged from shift planning for pharmacies, location and resource planning for emergency doctors and vaccination facilities, as well as the routing of ambulances. In particular, optimized plans were developed for regions around the German cities of Aachen, Erlangen, Kaiserslautern, and Nuremberg. Nevertheless, emphasis was put on the development of generic methodologies that can also be adapted to other regions as well. The research project, the particular case studies, the evaluation and validation of the developed approaches were performed in close cooperation with the respective domain experts who accompanied the project by providing historical data and their domain expertise. As a result of the project, a visualization platform was developed where results for the different case studies can easily be illustrated. Hence, the different stakeholders can see and evaluate various planning strategies. In more detail, the case studies considered here are the following.

We study the strategic question of where to erect vaccination centers in Germany in order to keep the travel distances acceptable while avoiding wasted vaccines and respecting the required number of locations and staff. Further, for optimizing the location structures of emergency doctors from a strategic point of view, it is necessary that all potential emergency locations can be reached quickly, while an optimal utilization of the available resources is maintained. From a tactical point of view, an important decision is to assign resources to the stations such that, in all scenarios, a sufficient amount of vehicles and staff is available to serve all occurring emergencies in an appropriate time frame. Further, for the planning of out-of-hours services of pharmacies, the main (tactical) decision is the assignment of out-of-hours services to pharmacies in such a way that these, for pharmacists unwelcome, services are fairly balanced over all pharmacies while distances for the population are still acceptable. Finally, an example for an operative planning question is reflected in the case study for the ambulatory care system, where transport vehicles must be routed under online updated information. The major goal here is to minimize the delays of patient transports, i.e. the waiting times of patients. Moreover, under Covid-19 requirements, the assignment of rides with infectious patients should be limited to only few vehicles, if applicable.

Besides merely developing optimization algorithms and handing out the results to the planners, in all of the above case studies, we also developed interactive software tools that make the solutions more comprehensible and tangible. For most analytic views, we developed cartographic representations. In this way, planners can compare alternative solutions, explore the winning and losing parties, and certify the feasibility of the results. Especially the latter is utmost important, as any deviation of a plan from its current standard has to be justified and defended in political committees. Hence, it is crucial that decision-makers are fully convinced of the advantages of their solution that necessarily represents a compromise. The goal of this article is to give an insight into different visualization tools that were implemented for the respective use cases and highlight their individual suitability for the respective purpose.

There is a vast amount of literature that studies mathematical optimization problems in healthcare services. Both exact as well as heuristic approaches have been presented and evaluated for healthcare applications in hospitals as well as in ambulatory medical care, and we refer to [2, 3] and the references therein for an overview. More generally, some absolute notes about bringing real world optimization questions of different planning scopes sustainably into practice can be found in [4]. Concerning the case studies presented here, we briefly review some of the related literature that investigates different aspects of the decision-support for healthcare systems. More specific related literature is mentioned in the according case study sections. For a recent overview on facility location problems in the healthcare sector we refer the reader to [5]. In [6], the authors provide concepts for planning the provision of emergency services. These concepts are currently applied by, e.g. the Ministry of the Interior and for Sport of Rhineland-Palatinate. However, the authors in [6] do not propose a decision-supporting tool and the focus of the presented model is more locally. Related work on planning of out-of-hours services for pharmacies can be found in [7–9], where the scheduling of duties in Turkey is considered. However, the modeling approach used there does not fit the needs of our practice partners, as elaborated in the corresponding “Case description” section. In our work, we instead use the basic model and algorithms described in [10]. Compared to [10], we provide an additional contribution here by focusing more on the aspects of a fair planning. An overview of routing approaches in healthcare logistics can be found in [11]. In this work, some transportation problems that arise in the healthcare sector of Austria are described, where the authors focus on ambulance services and the delivery of blood conserves.

The remainder of this work is structured as follows. In section “A street network framework for open map data”, we briefly present a general framework that we have used for computing the accessibility on real world street networks. Afterwards, in section “Case studies”, we present the different case studies by introducing the context, the arising planning questions, the optimization model and the decision support tools and maps. We start on the strategic level with the best-possible location of vaccination centers and emergency doctors in sections “Planning Covid-19 vaccine centers” and "Securing the provision of emergency services”, respectively. Subsequently, we present the tactical problem of improved pharmaceutical out-of-hours services in section “Securing the provision of pharmaceutical services”, followed by the operational task of optimizing the schedules of patient transports in section “Minimization of waiting time in patient transport logistics”. We discuss the challenges of transitioning from prototype software to production-ready tools in practice in section “Discussion” . We close with conclusions and further research questions in “Conclusions and outlook”.

## A street network framework for open map data

The considered case studies are all tightly connected to the access to medical care. Therefore, the reachability of various medical facilities (such as pharmacies or vaccination centers) is very important for the population. Conversely, it is even more important that ambulances and emergency doctors reach patients quickly. In order to compute realistic reachability times, a framework for a fast computation of shortest paths along such street networks is of relevance. Hence, we developed a street network data structure on which fast routing is possible. The framework is based on publicly available map data from *openstreetmap.org* (OSM, cf. [12]). A key feature of the data structure is that turning restrictions can either be respected or ignored when computing shortest paths. When considering vehicles within an emergency operation that are driving with sirens and thus have special privileges, the latter is a reasonable case to consider.

There are many applications that focus on routing or creating a data structure for street networks on map data retrieved from openstreetmap. To name a few of the existing tools, there are OSRM [13], osmnx [14], or graphhopper [15]. More routing engines for openstreetmap data can be found on its wiki page, cf. [16]. Most of the tools are, however, not easily included into other applications (due to license or compatibility issues). Others are rather heavy weighted: The amount of computational resources necessary for shortest path computations of larger areas of the map, such as the German federal state Rhineland-Palatinate, are too high to run the computations on a personal computer. Another downside of many of the tools is that they do not include turning restrictions in the routing process. Summing up, none of the existing tools completely satisfies our needs: A light weight framework, that efficiently computes shortest paths on reasonably large areas of the map, allowing the possibility to respect or ignore turning restrictions, and that can be included in different applications without too much effort.

In order to create a network from OSM map data, we first identify nodes that correspond to crossings and are therefore necessary for the network structure. In a second step, OSM ways are split at crossings. Finally, remaining crossings that are incident to exactly two streets with the same attribute sets are smoothed out to save further memory. In order to include turning restrictions, we choose from two possibilities: Either directly implement the turning restrictions into the graph or simply save the restrictions as a sequence of crossings and streets. In the first approach, the size of the network roughly quadruplicates and standard shortest path algorithms can be applied (for a survey on shortest path algorithms, cf. [17]). In the second approach, the network size remains unchanged but the shortest path algorithms need to be adjusted in order to ensure that shortest paths respect the turning restrictions. The adjusted shortest path problem is in general much more complicated than a standard shortest path problem. However, if the number of restrictions is low in comparison to the overall network size, the second approach proves to be more efficient. The source code for the tool that creates the described network and outputs it as geojson file can be found on [18].

For driving time estimations, the street network can be combined with an arbitrary speed profile, where a speed profile assigns each possible pair of street class and vehicle type an average speed. For most of our case studies, a common car speed profile is sufficient. However, for the presented case study of emergency doctors, the usual speed profiles are not adequate: Emergency doctors with sirens on are much faster and it is highly important to measure their response time accurately. Therefore, in that case, we estimate special speed profiles based on historical emergency operations data: We start with an initial speed profile for cars and calculate the fastest route for each emergency case. Given this route, we derive the driven distances on each street class. Together with the real driving time, which is known from the data, we estimate a new speed profile using robust linear regression. We repeat this procedure with the new speed profile until a suitable speed profile is achieved (cf. [19]).

## Case studies

In the following, we present four case studies that shall be exemplary for the various planning challenges planners in the ambulatory care system face in order to maintain or improve the medical care for the population.

The main difference is the scope of the four questions. For a strategic planning question, planners usually need to take high invest cost for the (re-)construction of infrastructure, e.g. buildings, into account. In contrast, tactical decision problems assume that basic investments are already fixed, but that resources (e.g. staff or vehicles) can still be reassigned with some flexibility. Strategical and tactical questions typically arise with some time in advance. Therefore, planning tools do not necessarily need to be highly integrated in the planners’ data and software environment. In the case of operational questions, decisions usually have immediate effects on machines or staff and therefore require a much deeper integration in order to work smoothly with online data updates. Consequently, also the frequency of decision-making varies. While operational decisions may occur every few seconds, tactical and strategical decisions are more likely to be made on a quarterly or even annual basis.

Another point of differentiation is the background and objectives of the planners. A professional chamber pursues other goals than politicians in the Ministry of Health or local districts which still differ from dedicated expert committees for healthcare optimizations.

Although the applications deal with rather different planning questions, the geographical aspect turns out to be important in all four of them. Therefore, while all implemented support tools use different kinds of visualization techniques, one key ingredient is an interactive map, where the planner can explore a larger region as a whole as well as the very local aspects. Additionally, this is a good way to convey relevant information in a compact manner. For more dynamic questions, animated simulation movies and retrospective Gantt charts have proven to be very helpful.

Ordered by their scope, we present the case studies in the following order:

1.: Optimizing Locations of Vaccine Centers for the Covid-19 Vaccines in Germany
2.: Optimizing Locations and Resources of Emergency Doctor Stations in Rhineland-Palatinate, Germany
3.: Optimizing Out-of-Hours Services for Pharmacies in the North Rhine Area, Germany
4.: Optimizing Patient Transport Schedules in Franconia, Germany

### Planning Covid-19 vaccine centers

#### Case description

In the anticipation of a vaccine against the novel coronavirus, which is expected to bring a rapid end to the Covid-19 pandemic, the Ministries of Health, together with expert groups from the federal and state governments, were faced with the question of where the population should be vaccinated. Hence, several scenarios were discussed: The common approach of letting all 55 000 general physicians administer the vaccine, using the ~400 health departments or only the 38 very specialized university hospitals. The decisions had to be made on the basis of a very uncertain data situation. Most of all, the number of vaccine doses available in the course of the vaccination campaigns and the actual vaccination readiness is still unclear. However, surveys from the COSMO study (c.f. [20]) have shown that the willingness drops significantly in case the one-way travel distance to the vaccination center exceeds 30 min. Further, reducing the wastage of vaccine doses is also a very crucial issue.

*Degrees of Freedom*

In summary, this results in the following decisions:

- Which of the possible vaccination centers shall be opened?
- How many physicians are needed in which vaccination center?
- Which citizens should be vaccinated at which vaccination center?

*Goals*

The objectives that need to be considered are:

- *Overhead Costs* Open as few vaccination centers as possible.
- *Patient Convenience* Keep the travel distances for the population as low as possible.
- *Waste Prevention* Use as few physicians as possible and reduce waste of vaccination doses as much as possible.
- *Robustness* The decision should not render invalid or become useless if the parameters differ from the expected values within certain ranges.

*Decision-Makers*

The decision-makers in Germany are the state-level health ministries. They are in professional exchange with each other and are interested in a consistent solution. Nevertheless, they have the sovereignty over planning within their respective states.

*Planning Scope*

The scope of the planning problem is mostly strategic, since the costs of implementing the vaccination infrastructure by far dominates the costs of shifting personal between centers.

*Status Quo Support Tools*

Prior to our engagement, to the best of our knowledge, no software tools have been used to answer these questions. Therefore, many internal discussions within expert committees apparently have been dominated by personal intuition rather than quantitative facts.

#### Mathematical optimization

We formulate the vaccination center planning problem as a linear Mixed Integer Program (MIP). Although MIPs are in general NP-hard problems^1^ , also large instances can nowadays be solved to global optimality by available state-of-the-art solution programs. For a general introduction to and overview of Integer Programming, we refer to [22]. To solve the integer program corresponding to our model we use Gurobi Optimizer, cf. [23]. The key idea of our model is the following. The decision variables model whether or not to open a vaccination center, the number of physicians assigned to the centers and the assignment of municipalities to centers. Depending on the scenarios, further constraints may enforce capacity restrictions or prefix assignments. For a comprehensive overview of location planning problems in the healthcare sector, see [5].

Since the planning on the national level has foremost been intended as a quantitative comparison of the presented scenarios, we refrain from planning on the level of street networks or detailed population distributions. Instead, we use linear distances between the optional vaccination centers and the center coordinate of the German municipalities. More details on this particular case study can be found in [24].

#### Decision support tools

**Fig. 1:**
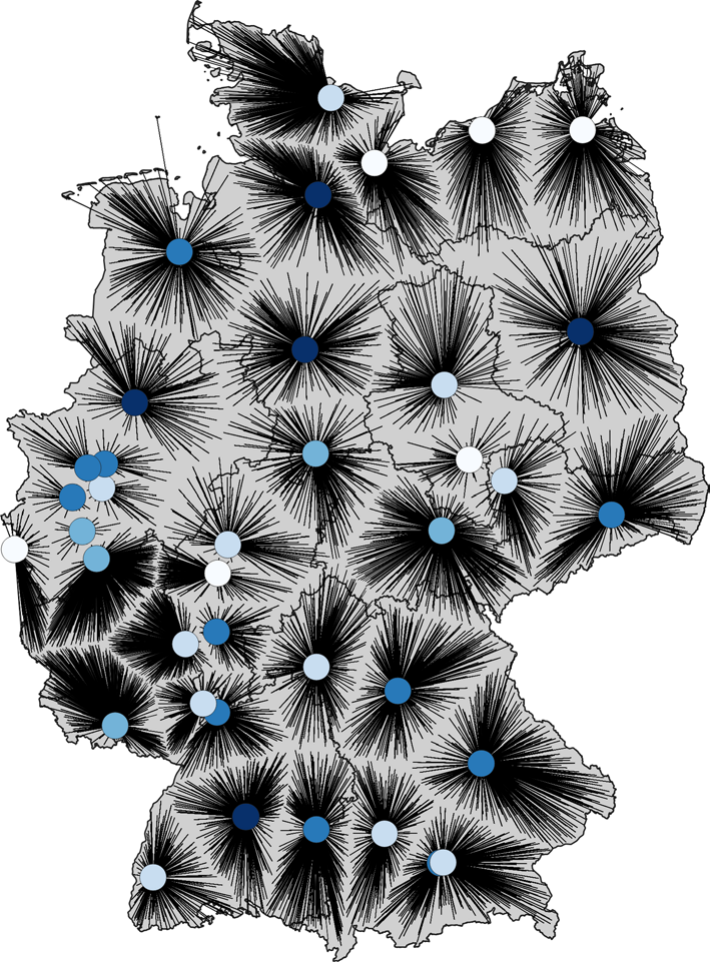
Example for an Allocation Chart for the university scenario.

**Fig. 2:**
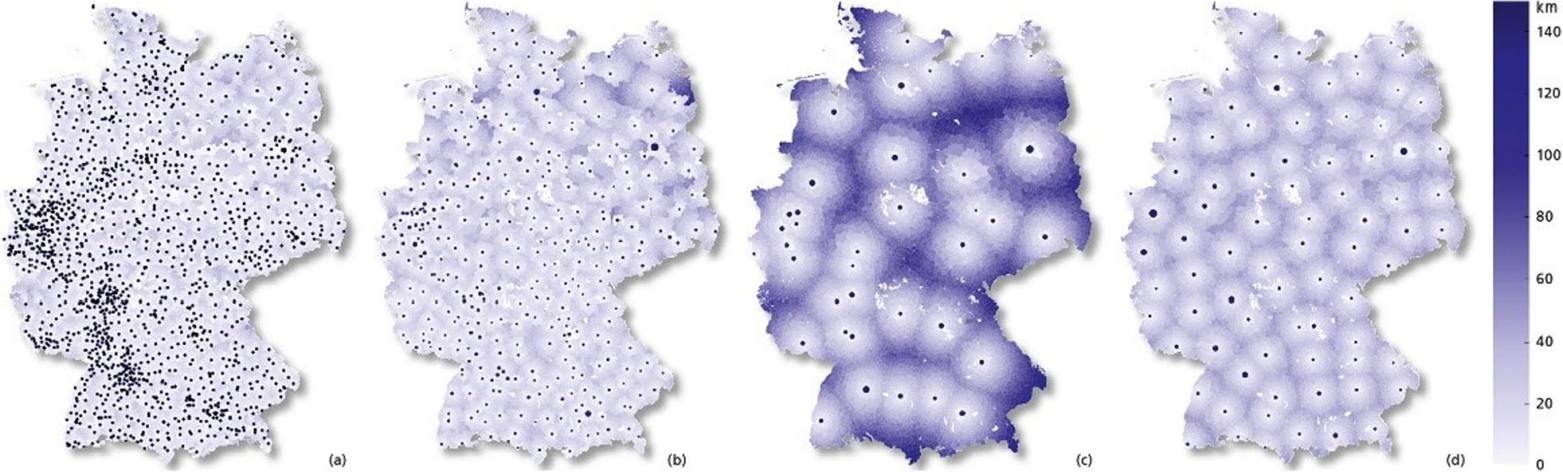
Example for Choropleth Maps for different scenarios. **a** Vaccination at physicians, **b** Vaccination at health departements, **c** Vaccination at university hospitals, **d** Vaccination locations based on green field planning

Although the question posed in this case study is of a more strategic nature, the project was still characterized by the need of an urgent answer. We have focused on the following visualizations:

1. *Visualization of assignments by Allocation Charts* In an Allocation Chart, we position the (vaccination) sites as markers (circles in this case) on a map and connect all assigned regions with a straight line. An example is shown in Fig. 1. Using this visualization, the planner can easily see which communities are assigned to which vaccination centers. Here, we are creating mostly way-optimized scenarios in the sense that lines usually do not cross. Hence, this visualization makes it easy to understand the structure of the assignment and compare it with other strategies. The colored markers can additionally be used to represent the total number of assigned patients/physicians per vaccination center. Note that, when used in a vector format, this representation is not restricted to the university hospital scenario, but can also be analyzed interactively on a regional level for scenarios with far more locations.
2. *Visualization of travel distances by Choropleth distance maps* Choropleth maps are thematic maps where geographical regions are colored according to a categorical value or a statistical variable which is associated with this area. This visualization technique has been used for almost 200 years and is still a very popular method of visualizing data (cf. [25]). However, it is not possible to color a district with respect to a distribution or multiple values at the same time. For the present case study, we have colored the German municipalities according to their median travel distances in the respective scenarios, since this value is both meaningful and comprehensible to the planners. Any other quantile would work likewise, but may be harder to grasp. A result example for different scenarios of location strategies can be found in Fig. 2. Here, we use a continuous color scale. Traditionally, Choropleth maps use discrete color schemes. For many scenarios, the assignment of municipalities to vaccine centers have been prefixed by definition. In that case, the median in fact represents the total population’s travel distance. We also use the center markers’ sizes to represent the number of physicians assigned to a vaccination center.

#### Challenges and outcome

The study results have been presented to the Robert Koch Institute (RKI), a German federal government agency and research institute responsible for disease control and prevention. The vaccination experts forwarded the results to the decision-making bodies. We received the positive feedback, that the results are interesting and will be taken into consideration for the actual vaccination center planning. However, as other goals, which are not easily included into our models (e.g. political motivations), are also of interest to the authorities, our proposed solutions were not applied directly and rather gave a rough idea on which travel times and necessary capacities result from a certain number of centers in Germany. Ultimately, a vaccination center strategy similar to the one presented in Fig. 2b has been realized in Germany.

### Securing the provision of emergency services

#### Case description

In Germany, emergency services are based on two pillars, ambulances and emergency doctors. While ambulances are sent out for all emergencies requiring medical assistance, emergency doctors are only dispatched if the current emergency is severe and in need of an actual doctor. The emergency doctors are then taken to the emergencies in vehicles separate from the ambulances. This system is commonly referred to as the *rendezvous system* [6]. In this case study, we focus on the strategic planning of placements of emergency stations and the corresponding tactical provision planning of emergency vehicles and the according staff. The planning must respect the conflicting goals of providing sufficient care in terms of response times and budget. In the following. we summarize the case description in a condensed form.

*Degrees of Freedom*

- Where should emergency stations be opened?
- How many emergency doctors should be on duty in what station at which time?

*Goals*

- *Covering residents* Provide sufficient care for the population, i.e. minimize patient’s waiting time for emergency care.
- *One-Time Costs* Open as few emergency stations as possible.
- *Operational Costs* Reduce number of doctors on duty as much as possible.

*Decision-Maker*

The federal ministries of the states are responsible for planning the provisions of the emergency services.

*Planning Scope*

The scope of the planning problem is both strategic and tactical, since long-term plans for a location’s structure induce large investment costs and cannot be altered easily. The provision of emergency services are easier to adapt later on. However, ideally, a planning considers both aspects in an integrated way.

*Status Quo Support Tools*

Prior to our engagement, to the best of our knowledge, the ministries planned the location structure and provision manually. Some ministries make use of a statistical approach based on the Poisson-distribution by Betzler et al. (cf. [6]). In this approach each emergency station is regarded individually and the map has to be partitioned manually (or by any method of choice) into regions for which a single station is responsible. The downside of this approach is that in actual emergency cases, it may very well be possible that the closest, or responsible, vehicle is not available (even if, by the approach of [6], the probability of such an event is kept small). This effect is commonly referred to as *duplicity*. Further, a visualization tool for ambulance service planning is given in [26] which allows to analyze the significance of each ambulance station but does not incorporate an optimization model for provision planning.

#### Mathematical optimization

The basic mathematical model for the integrated location and provision planning, called *q*-*Multiset Multicover*, has been published in [27]. In this model, we are given a set of possible locations and a set of regions to be covered, where each region is assigned an integral number of *clients*. Further, we are given an incidence relation between the locations and regions. A solution to *q*-*Multiset Multicover* is an allocation of suppliers to the possible regions such that all clients can be served, where each supplier is assumed to be able to serve up to *q* clients in its neighboring regions. The objective is to minimize the number of required suppliers. In the robust version of *q*-*Multiset Multicover* the number of clients in each region is subject to uncertainty and we aim to ensure feasibility for all scenarios of a given uncertainty set. A single scenario in this case is one possible distribution of clients to the regions of the instance. The basic model has been extended and adjusted to fit the location and provision planning for emergency doctors in a robust fashion based on historical emergency data and street network data from *open street map*.

#### Decision support tools

In order to make the optimized provision plans more tangible for the users, we have developed a software tool that allows to explore plans interactively. The main decision support tools are the following.

**Fig. 3:**
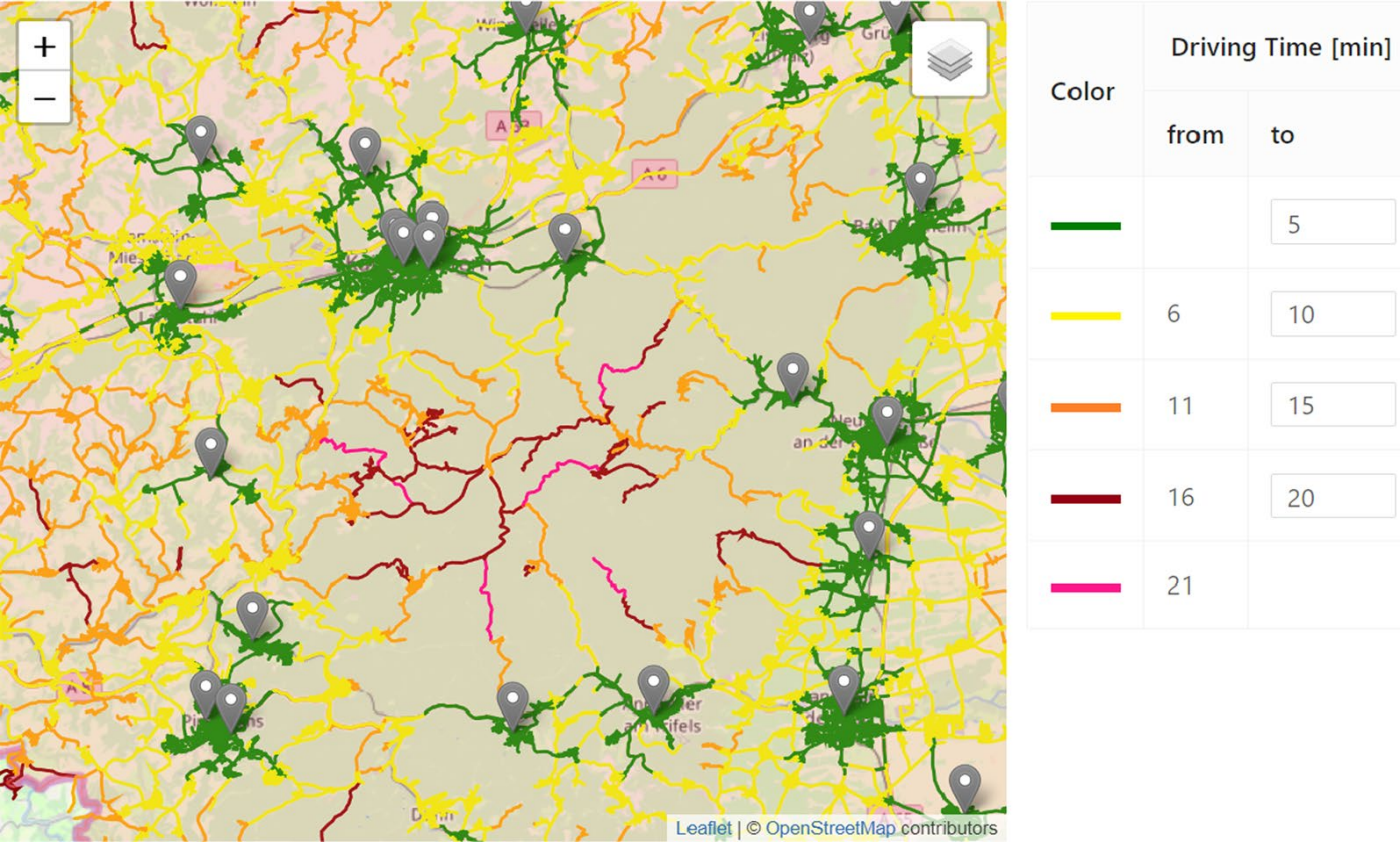
*Isochrone Map:* Street network colored according to the estimated driving times from the closest station

**Fig. 4:**
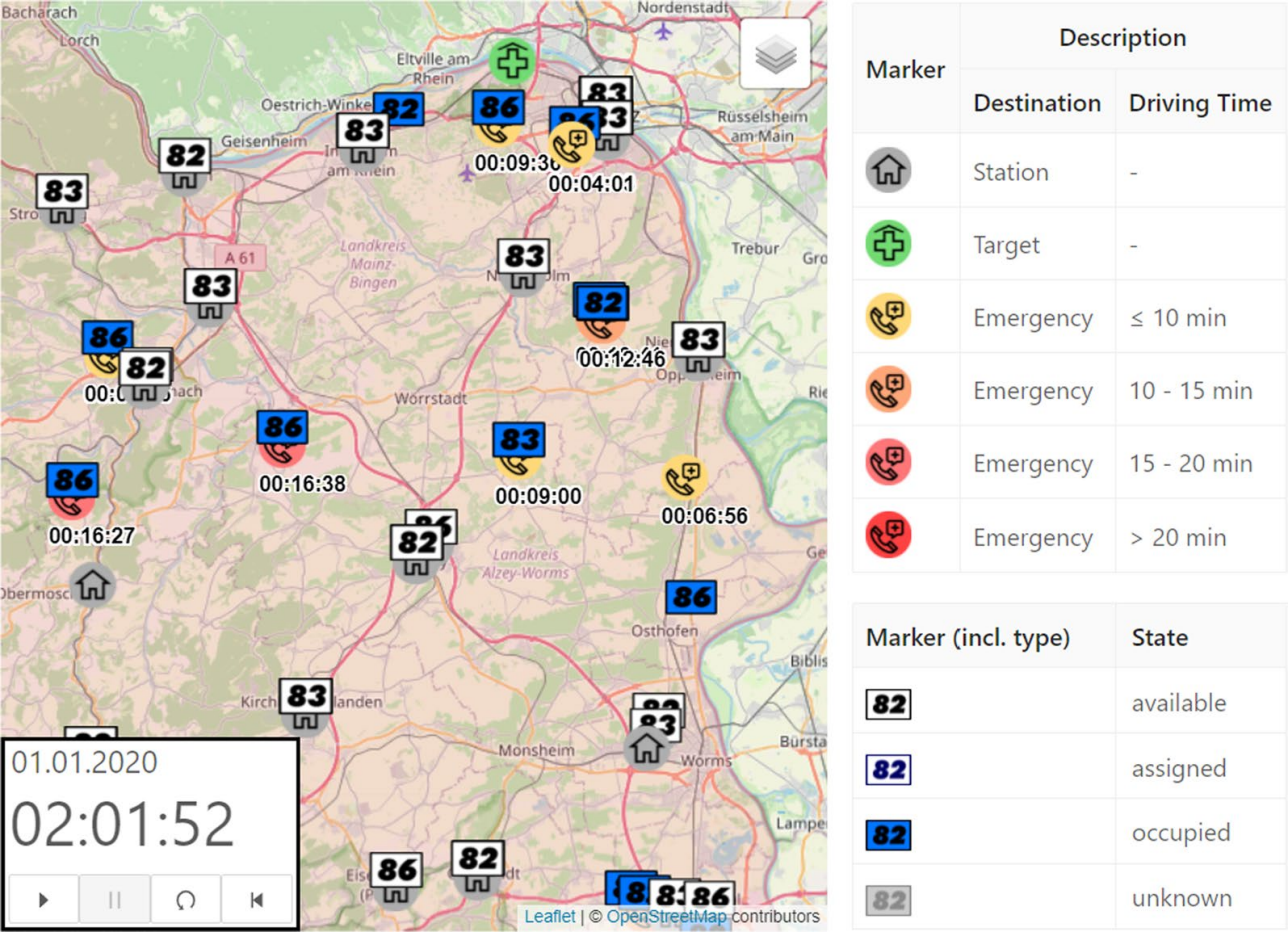
Snapshot of the dynamic event-based Simulation Animation

**Fig. 5:**
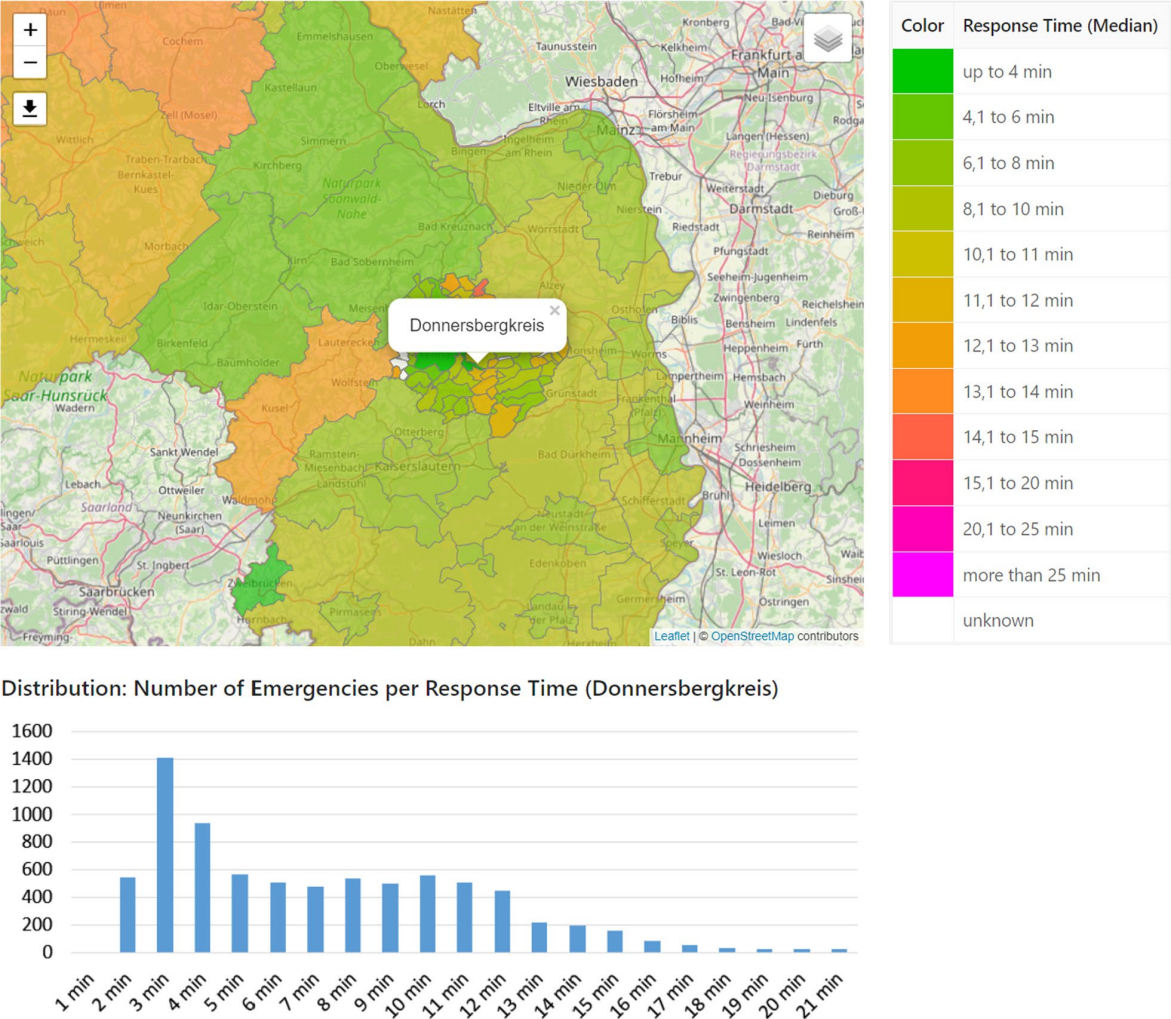
Exemplary evaluation of the median response time within a county or municipality for fictitious data

1. *Isochrone Map* Coloring isochrones are a common method to visualize destinations that can be reached within the same amount of time from a certain origin (cf. [28]). By superposing the isochrones of all opened emergency stations, the decision-maker can easily spot regions that no emergency vehicle will reach within a desirable time, when starting at the station. However, in order to have meaningful isochrones in the specific application, it is crucial to use accurate time metrics for the street network (cf. “A street network framework for open map data”): We have implemented a street network data structure based on map data from *open street map*, that distinguishes between turning restrictions which can be violated by a an emergency vehicle and those that are enforced by structural separations (cf. [18]). Further, we have created a data based driving profile for blue light drives using a robust linear regression (cf. [19]). As shown in Fig. 3, we color the street segments according to the computed distances, where red and pink segments highlight regions which cannot be reached in a short time from the emergency stations.
2. *Simulation Animation* In general, emergency physicians do not necessarily start at the station, but are already handling other cases, at the time they get alerted. If they are available, they will drive directly to the next location. Therefore, response times can be shorter or larger than in the static analysis. Due to the possible simultaneous occurrence of several emergencies, waiting times may further increase and especially depend on the shift strength. We have implemented an event-based simulation which can simulate and evaluate the response times of historical emergencies under varying (optimized) provision plans. A description of a similar simulation framework (not based on OSM data) can be found in [19]. We have also implemented a dynamic visualization, where the emergency vehicles can virtually be tracked on a map in real-time or arbitrary speed and the patients’ waiting times are highlighted. This visualization helps the planners to gain trust in the simulation data and allows for an in-depth analysis of certain situations. Figure 4 shows a snapshot of the simulation visualization where one can see white and blue ambulances and rescue vehicles (indicated by different numbers). White vehicles are available at the station (gray marker) or move back to the station, while they can be alarmed. Blue vehicles are currently occupied and moving to an emergency scene or care for a patient. Emergency scenes are visualized as yellow and red markers where the color depends on the waiting time which counts up below the marker. Green markers symbolize the destination of a patient transport, e.g. hospitals.
3. *Median response time analysis* The results of the simulation runs can also be evaluated in an aggregated form. We have therefore again used choropleth maps that visualize the median response times of the historical emergencies on county level. For more detailed insights, the response times can also be analyzed on municipality level and the distribution of data is shown as a histogram chart. Figure 5 illustrates this analysis for a fictitious scenario.

#### Challenges and outcome

One of the major challenges during the case study was the handling of the historical emergency data, which contained many erroneous data sets and, due to data privacy reasons, it was hard to get a hold of it in the first place. However, with the help of expert feedback, we were able to create guidelines to recognize relevant emergency missions and implemented decision rules to filter the data accordingly. Further, the Ministry of the Interior and for Sport of Rhineland-Palatinate was happy to confirm many of our findings. For example, they stated that the isochrone map (created with our speed profiles) was in line with their intuitive idea of reachability in the street network of Rhineland-Palatinate. The developed tools are currently in an alpha version which is not directly applicable for practical usage. Due to the mentioned positive feedback, the Ministry of the Interior and for Sport of Rhineland-Palatinate decided to fund the project ONE PLAN in which the strategic planning as well as the event-based simulation is brought to application maturity.

### Securing the provision of pharmaceutical services

#### Case description

The continuous access to pharmaceuticals is fundamental to the functioning of health care systems. In Germany, this access is—during day and night—mainly ensured by pharmacies. Therefore, every day a subset of pharmacies performs out-of-hours services, which are essentially 24 hours shifts. This subset of so-called out-of-hours pharmacies varies on a daily basis and is specified by the responsible chamber of pharmacists.

Performing out-of-hours services is an expensive burden for most pharmacies: A highly qualified pharmacist must be present during the entire 24 hours shift, while the demand for pharmaceuticals is usually relatively low at night. Hence, on the one hand, most pharmacies would like to perform as few out-of-hours services as possible. On the other hand, the out-of-hours service is crucial for the health of residents. This implies the necessity of an efficient planning of out-of-hours services that guarantees an adequate coverage of residents and minimizes the burden on pharmacists.

*Degrees of Freedom*

- Which pharmacies are assigned an out-of-hours service on which days of the year?

*Goals*

The objective is to minimize the total number of out-of-hours services while the following requirements need to be considered:

- *Covering Residents* For every day of the year, a prespecified number of out-of-hours pharmacies must be *reachable* from the center of each municipality.
- *Geographic Dispersion* Pharmacies that are in *vicinity* of each other must not perform an out-of-hours service on the same day.
- *Periods of Rest* Between two consecutive out-of-hours services of the same pharmacy, there must be a prespecified period of rest.
- *Equitable Distribution* The burden of out-of-hours services should be distributed in a *fair* manner among pharmacies.

Note that *reachability* for the coverage and *vicinity* for the dispersion can be defined with respect to distance in the street network or travel time considering different types of transportation. Here, we consider the distance in the street network, which is in accordance with regulations of the Chamber of Pharmacists North Rhine, one of our practical partners. From a mathematical perspective, our models support all metrics as long as the required data is available. The definition of a *fair* distribution is less straight-forward since its interpretation strongly depends on personal viewpoints. Moreover, each interpretation has to be modelled differently. We will further discuss this requirement of *Equitable Distribution* in the “Mathematical optimization” section below.

*Decision-Makers*

The decision-makers in Germany are the 17 chambers of pharmacists, which are commissioned by the federal states with the public supply mandate and are legally obliged to ensure that every resident can find an open pharmacy 24/7 within an appropriate distance (cf. [29]).

*Planning Scope*

The scope of the planning problem is tactical, although out-of-hours-plans are determined once per year, one year in advance. The planning problem does not, in particular, entail long-term decisions and plans can be adjusted if necessary.

*Status Quo Support Tools*

Currently, the planning areas of most German chambers of pharmacists are divided into districts, in which the out-of-hours service is organized locally in a rotation system (cf. [30]). This has the advantage that the decision-makers have a solid understanding of the local situation.

However, a decentralized planning is usually less efficient compared to a holistic approach, as the out-of-hours plan is not synchronized across the different local districts. In [7–9], the problem of synchronizing the rotations within the given districts in order to optimize the coverage of residents is considered. A downside of this approach is that the practicability of the resulting plan depends on the given division into local districts. On the one hand, the plan might not provide sufficient coverage of the residents if the local districts are too large. On the other hand, the burden on the pharmacists might be too high if a district contains few pharmacies. In Germany, the division of pharmacies into local districts has already led to legal actions by pharmacies against the chambers of pharmacists in the past (compare VG Sigmaringen, judgement of October 25th 2005, sign 9 K 284/04). The main reason for concern is the fact that the number of pharmacies per district may vary, leading to vastly uneven numbers of annual out-of-hours services.

To address these issues, the Chamber of Pharmacists North Rhine, which was formerly divided into 69 local districts, performs a centralized algorithmic planning since the beginning of 2014 (cf. [31]).

For this, planners use a tailored decision support tool that can provide heuristic out-of-hours plans as well as textual summaries. Thus, there is still potential for further improvement when using visualization and mathematical optimization. Visual data summaries, e.g. in the form of maps, can aid and improve the complex decision making and validation process. Algorithmically, we can not only try to reduce the number of out-of-hours services, but emphasize the satisfaction of the planning constraints. The currently used algorithm, which can be seen as a greedy heuristic, regularly needs to relax some constraints, which is not surprising, as it is already NP-hard to construct any feasible plan (cf. [10]). Especially abrupt changes of the status quo, such as in the Covid-19 pandemic, overstrain the greedy planning heuristic as additional constraints, e.g. for computing a robust plan, need to be considered.

#### Mathematical optimization

Formalizing the objectives and requirements for the planning of out-of-hours services for pharmacies, we obtain a MIP that can yield feasible plans with a minimum number of out-of-hours services (cf [10]).

As mentioned in the “Case description” section above, the definition of fairness as a key requirement is generally difficult to measure and nontrivial to formalize due to its strong dependence on personal viewpoints. Since we want to distribute the burden on the pharmacies evenly, we measure fairness with respect to the number of services a pharmacy has to perform compared to other pharmacies. A very basic approach to achieve fairness would be to allocate the same number of out-of-hours services to all pharmacies. However, while the burden of the services can be distributed easily within cities, that have a high pharmacy-density, pharmacies in rural areas often have to perform more services in order to guarantee the coverage of residents. Enforcing an equal number of services for all pharmacies thus leads to an unnecessary high number of services within cities. We must therefore distinguish between pharmacies of different regional specifics to obtain an efficient plan. Discussions with the Chamber of Pharmacists North Rhine revealed that they consider a plan to be fair if all pharmacies within the same municipality perform roughly the same number of services. Unfortunately, enforcing equity on municipality level can have undesired implications regarding global fairness when computing plans that are optimal regarding the total number of assigned out-of-hours services: If a municipality contains several pharmacies that are less valuable with respect to our coverage model, it may be most efficient to assign no out-of-hours service to any pharmacy within the municipality. We address this problem in two different ways. First, as every pharmacy is legally obliged to participate in the out-of-hours service (cf. [32]), we enforce a minimum number of annual services for each pharmacy. Second, we limit the difference in the number of services that pharmacies in neighboring municipalities can have. For this, we consider a simplified planning problem, for which we compute an idealized plan using an efficient greedy algorithm. This plan fulfills the lexicographic fairness property, which is considered as the most equitable concept for the distribution of loads by some studies (cf. [33]). The relation of the number of out-of-hours services assigned to pharmacies within neighboring municipalities in the idealized plan then defines the relation of the services in the real plan.

Finally, it must be noted that due to its size and practical difficulty, the previously described exact MIP becomes intractable for the real-world planning problem of the Chamber of Pharmacists North Rhine. We therefore developed an exact reformulation via aggregation of mathematically equivalent pharmacies. This aggregation significantly reduces the size of problem instances, while simultaneously breaking symmetries that represent a common challenge in large-scale mathematical programming (cf. [34]). Moreover, we introduced a time-based decomposition of the planning horizon by employing the rolling horizon paradigm. The resulting solution approach is no longer exact, but yields a significant speed-up of the solution process and close-to-optimal solutions for the large scale instances that are derived from our practical application (cf. [10]).

A major advantage of our approach is its flexibility for testing new planning concepts. We are able to add new planning constraints and evaluate their implications. For example, a report was provided to the Chamber of Pharmacists regarding a closer coverage of medical practices by out-of-hours pharmacies. The exactness of our approach allows us to determine which additional burden would be implied by different coverage parameters. With respect to the Covid-19 requirements, we further extended all models by a robustness measure that requires plans to remain feasible if no more than a prespecified number of pharmacies cease operation due to quarantine measures.

#### Decision support tools

Visualization is instrumental to the analysis and evaluation of out-of-hours plans. To facilitate this process, the implemented decision support platform includes various visualizations such that users can easily analyze and verify out-of-hours plans with their expert knowledge.

**Fig. 6:**
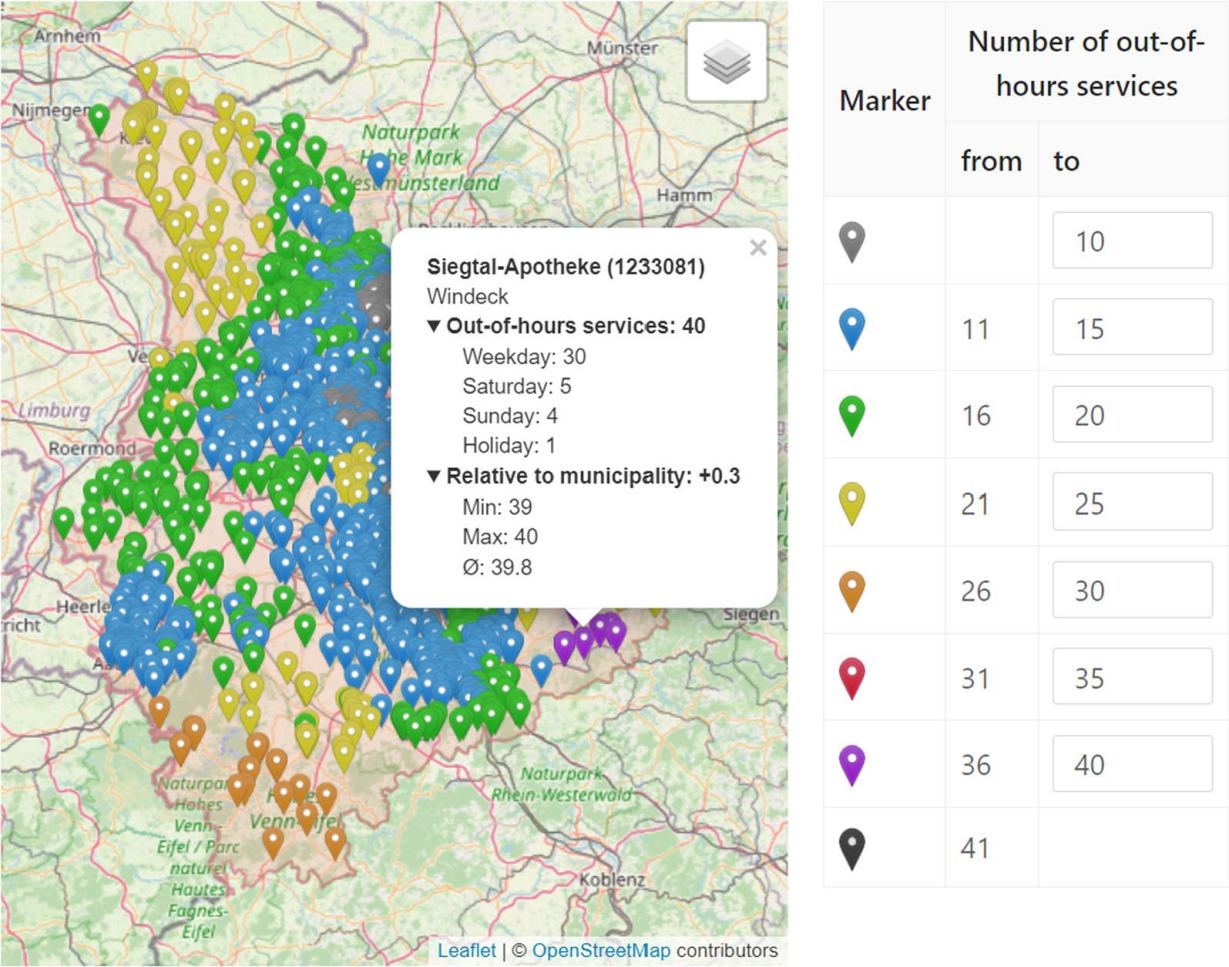
*Marker map*: Out-of-hours services per pharmacy with compact detail information. The user can customize the intervals

**Fig. 7:**
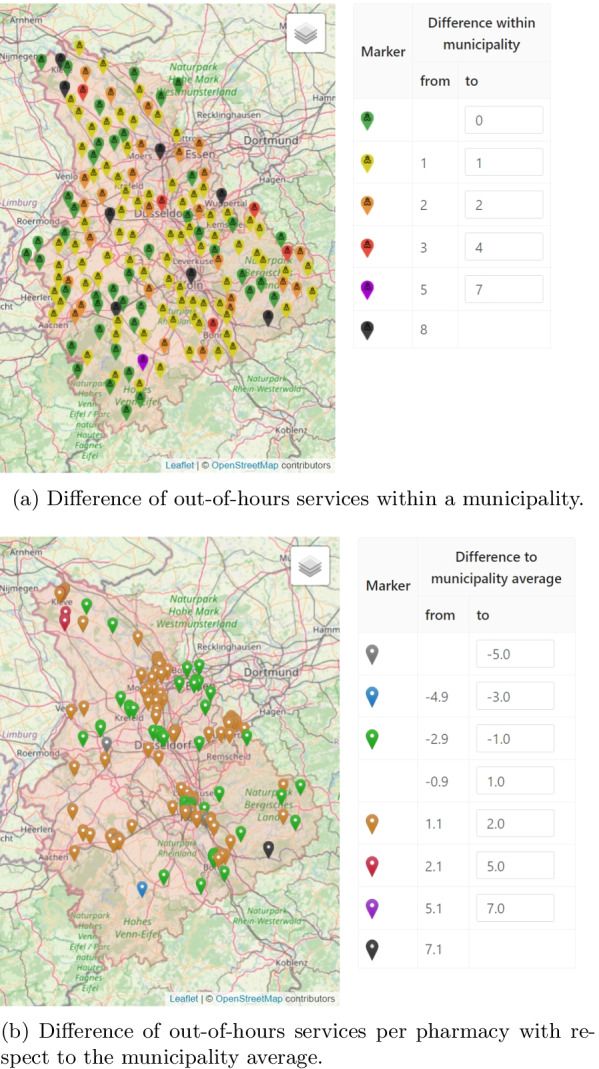
Fairness charts

**Fig. 8:**
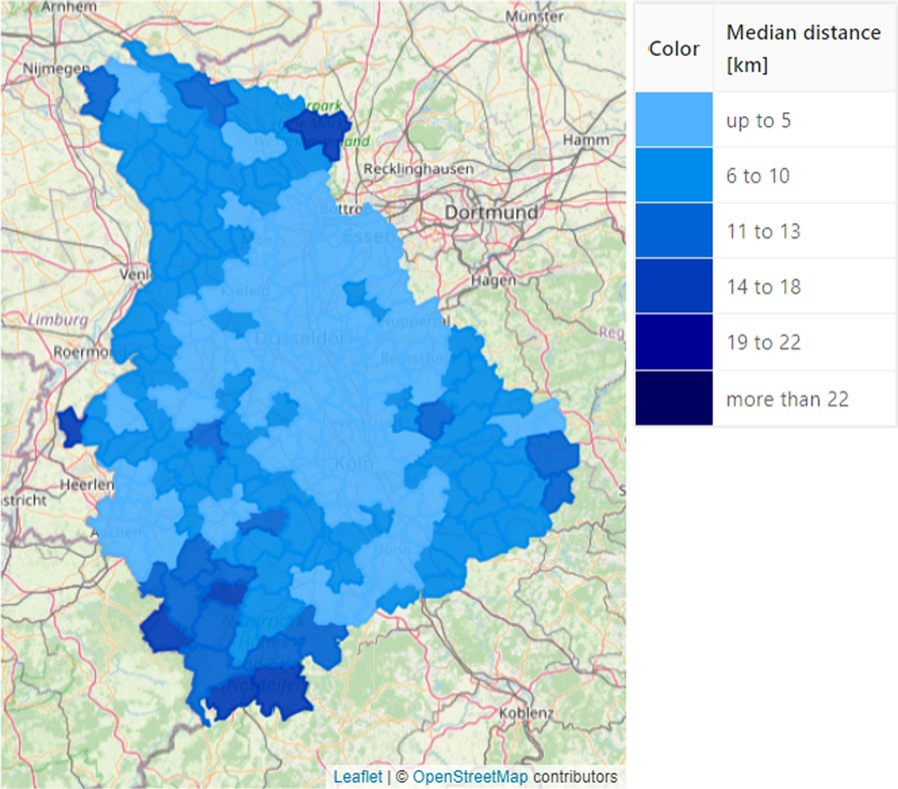
*Reachability chart*: Municipalities colored according to the median driving distance to the next out-of-hours pharmacy over a year

1. *Marker Maps*
  - *Duty Charts* As stated above, a key goal is to minimize the number of out-of-hours-services per pharmacy. We therefore implemented a view that highlights the individual load of each pharmacy in form of a *Marker Map*, cf. Fig. 6. Here, each pharmacy is represented by a pin, which is colored with respect to a performance indicator, e.g. the total number services in the current plan. The coloring intervals can be modified by the user. A tooltip provides further detailed information (distribution of out-of-hours service numbers and their comparison to the municipality) in a compact form. Other performance indicators that can be visualized are, for example, the difference of services in an optimized plan with respect to the original plan. In contrast to providing the information merely in tables, systematic effects can easily be spotted on the map. Examples are the increased load in rural areas or at boundaries as shown in Fig. 6.
  - *Fairness Charts* Another example for a marker map are fairness charts (Fig. 7). In the municipality view (Fig. 7a), we represent each municipality with a marker that is color coded according to the maximum difference in the number of services that the respective pharmacies have to perform. In the pharmacy view (Fig. 7b), we again represent pharmacies by pins and visualize each pharmacy’s difference to the average number of services within its municipality. This view accounts for the fact that pharmacists accept that their workload differs due to structural reasons, however they do not want to be disadvantaged compared to their colleagues from the same municipality.
2. *Reachability Chart* In addition to fairness, the reachability of the population is an important criterion when creating an out-of-hours plan. For this reason, we developed a reachability analysis on a daily and annual basis. For the former, we calculate the shortest distance from an open out-of-hours pharmacy to each street section. Then, we color the street network according to the calculated distance from an open pharmacy. Recall that  this method is explained in more detail in the Decision Support Tools section of the second use case where an example for emergency doctors is shown in Fig. 3. For pharmacies, however, this analysis is only a snapshot as out-of-hours pharmacies change on a daily basis. Therefore, we additionally calculate the shortest distance from an open pharmacy to each municipality throughout the year. Due to computational effort, we only consider the distance to the municipality center instead of each road segment. Figure 8 shows all municipalities colored according to the median distance to the closest out-of-hours pharmacy over the year. It is easy to see that the access distances are greater in rural and peripheral areas, although pharmacies in these regions already offer significantly more services, as shown in Fig. 6. The possibility to visualize this trade-off between reachability and load on the pharmacists enables the decision-makers to analyse such problematic regions more thoroughly. The parameters and constraints of the optimization model can then be adapted to react to an unsatisfactory coverage of the residents or an overburdening of the pharmacists.

#### Challenges and outcome

In our research, we developed methods for optimizing out-of-hours service plans and an interactive visualization platform so that planners can quickly assess them.

A major challenge in developing the optimization methods was finding a tractable definition of fairness that burdens similar pharmacies similarly, but provides the freedom to treat pharmacies in different areas individually for the sake of efficiency. The main obstacle for deploying the resulting optimization method is the need for continuous support in the day-to-day planning process, which usually cannot be guaranteed by scientific partners. Therefore, the Chamber of Pharmacists North Rhine decided to stick with their current planning tool. While their tool needs to relax planning constraints and assigns more services compared to our methods, it still produces sufficiently good plans.

Nevertheless, the developed optimization methods were valuable in several consultation processes. As already mentioned in the above “Mathematical optimization” section, we aided the Chamber of Pharmacists North Rhine in deciding whether to implement new concepts of covering residents. The interactive visualization platform played a critical part in these consultations, as it allows for a quick evaluation of the generated out-of-hours plans. At the request of the Chamber of Pharmacists North Rhine, the platform was made available for their day-to-day business. The software is hosted online with restricted access and is currently reviewed by the Chamber of Pharmacists North Rhine.

### Minimization of waiting time in patient transport logistics

#### Case description

In Germany, the transport of patients to or from hospitals and medical practices is not only done with rescue transports in case of an emergency, but also with patient transports for less urgent cases. Examples are regular dialysis appointments, other hospital appointments or discharges for patients that cannot provide other means of transportation. Many patient transports are known at least one day before they take place, making them *plannable*. However, the dispatcher is also called, e.g., by hospitals for transporting a currently released patient. This results in an *ad-hoc transport* request for the near future which needs to be scheduled on the fly and may disturb the original schedule. Even the known transports may not be performed as planned in case driving or handling takes longer than estimated. Hence transports need to be assigned to patients under incomplete information. In order to keep the waiting time for all patients as low and foreseeable as possible while still carrying out all transports, creating plans that take these imponderables into account and respect shift times is reasonable.

During the Covid-19 pandemic, further requirements have to be taken into account. If the transported patient is infected with Covid-19, there is a nonnegligible risk of infection for employees and following patients. To lower the risk of infection, drivers and medicals have to wear particular protective clothes if a patient is known to be infected. Additional to the usual disinfection time of a vehicle, it takes time to put these clothes on and off before and after a transport. Apart from this extra effort, the number of protective clothes is limited—and they have to be changed after each transport. Thus, these additional constraints have to be taken into account as well. Tasks related to the patient transport problem described here are presented, for instance, in [35, 36]. In the first one, the authors model the patient transport problems in the Netherlands. The main difference is that there is no distinction between emergency and patient transports as it is in Germany. In the latter and more recent one, the authors describe a similar problem in the US—but in a stochastic version, i.e., the duration and travel times can be random variables. An application during the Covid-19 pandemic is given in [37]. Therein, the authors discuss the distribution of face shields in Spain. This is solved heuristically using a vehicle routing model.

*Degrees of Freedom*

- How are the patients scheduled to the transport vehicles?
- At which times are patients fetched?

*Goals*

- *Patient comfort* The waiting times for patients should be kept as low as possible.
- *Fairness* The waiting times for patients should be balanced in such a way, that no patient should have to wait a disproportionately long time.
- *Robustness of the plan* Ad-hoc transports, that only reveal themselves during the day of action, should not delay the transports of other patients, if possible.
- *Labor Protection Laws* Drivers are not supposed to work over-due, i.e., the plan must respect the shifts.
- *Handling of possible infections* Transporting infected patients should neither endanger other patients nor lead to huge extra efforts.

*Decision-Makers*

In Germany, the scheduling of patient transports is distributed to smaller subareas. In our considered region, the dispatcher is the control center *Integrierte Leitstelle Nürnberg*. In addition to patient transports, it is also responsible for dispatching fire and rescue transports. All these transports generally do not affect each other because they have different fleets of vehicles and tasks to perform.

*Planning Scope*

The scope of this planning problem is operational, since the decisions immediately affect the patients and drivers. Also, due to the ad-hoc transports, the planning must be performed in real time and plans are altered multiple times a day.

*Status Quo Support Tools*

Prior to our engagement, to the best of our knowledge, no software tools had been used to answer these questions. This mainly relies on intuitive predisposition. At the moment, the scheduling method of the control center dispatches rescue transport vehicles, patient transport vehicles and firetrucks in the same way, namely by dispatching the nearest available vehicle to a requested transport.

#### Mathematical optimization

The basic model we use is the *Vehicle Routing Problem with General Time Windows* (VRPGTW) [38]. For this problem, one is given a fixed amount of customers that have to be served by a fleet of vehicles. The vehicles start from a fixed depot and have to finish their route in their depot. Each customer has to be served exactly once and has an additional time window in which the service is supposed to be carried out. If it is a so-called *hard* time window, service necessarily has to take place within the window. For a so-called *soft* time window, service can, but should not, be outside the window. For optimized schedules, both the travel time to reach a customer and the duration of a service have to be taken into account. In the considered case study, patients need to be transported to a certain point in time that is modeled as a soft time window, since a delay is allowed, but naturally not desired. The drivers’ shifts correspond to hard time windows for the vehicles. At the beginning of each shift, a vehicle is stationed at its depot, where it has to return to by the end of the shift. Meanwhile, it can handle an arbitrary number of transports, each consisting of a start and an end position and a time at which the patient needs to be picked up. Every transport has to be handled by exactly one vehicle, preferably exactly at the scheduled time. As this is usually not possible, the goal is to find a schedule that minimizes the waiting time, i.e., the amount of time a service starts later than originally scheduled.

For reasons of fairness, the maximum waiting time is minimized first, and secondly the overall delay over the day. Our VRPGTW instances are modeled as MIPs that can be solved efficiently with available state-of-the-art solution program. A MIP is solved once for all plannable transports at the beginning of the day. Whenever a new ad-hoc transport is requested, the problem is re-solved. Already started or finished transports are fixed in that calculation, others can be rescheduled.

When taking Covid-19 transports into account, the service time needs to be adjusted by the additional time for putting on and off protective clothes or disinfecting the vehicle. In particular, when the total number of Covid-19 cases is relatively low, separating known Covid-19 transports from others, whenever possible, is advantageous. Thus, keeping different fleets of vehicles with only a small number of *floater* vehicles which can transport any patient is an appropriate course of action. The VRPGTW model is adjusted accordingly. For more details on the solution approach for patient transports, the Covid-19 specific modifications and computational results, we refer to [39].

#### Decision support tools

**Fig. 9:**
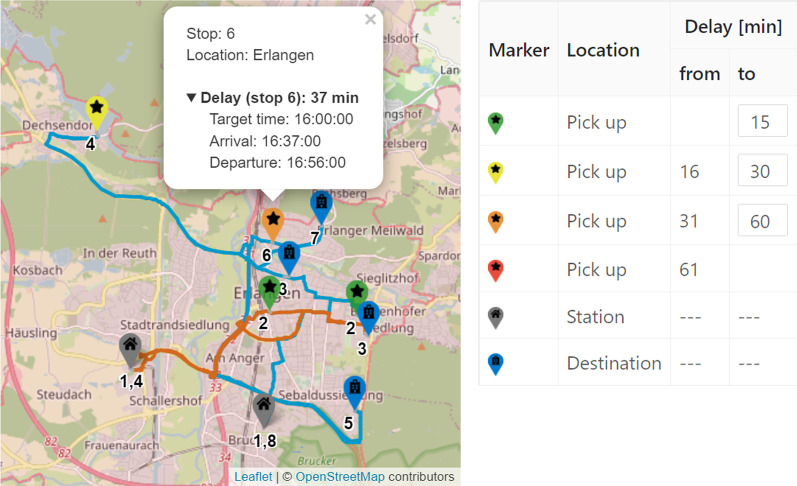
Example for a Route Map with two routes with six stops each

**Fig. 10:**
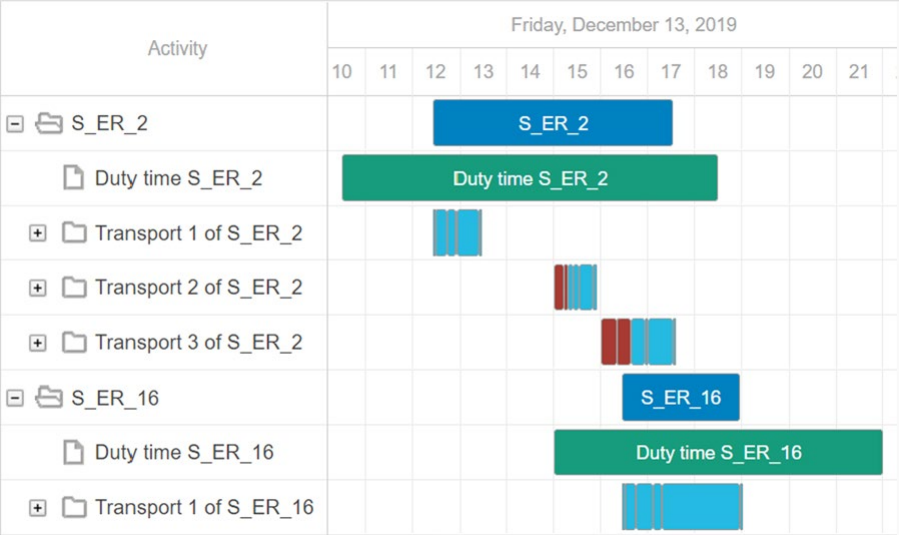
*Gantt Chart* representation of the transport activities from Fig. 9

1. *Route Maps* Since the triumph of map-based navigation devices, the display of routes on maps is familiar to everyone. When retrospectively analyzing the routes of the transport vehicles, a route map is helpful to clarify the geographical distribution and distances to the viewer. In our decision support tool, the user can display and follow the daily routes of selected vehicles on a map. Since the exact GPS paths of the historical transport routes are not available, we compute the shortest routes between the known locations on the OSM-street network (cf. “A street network framework for open map data”). The stops on the route are represented by different markers. The start and end point of a daily trip is the depot (grey marker with house). In between, pick up locations (green, yellow, orange, red markers with star) and destinations (blue marker with hospital) are alternately approached. The color of the pick up location marker depends on the delay at that time. Figure 9 shows an example of the daily routes of two vehicles. Here, the user can see at a glance for which trips delays have been built up or reduced. By clicking on the individual markers, detailed information about the stop, such as target time and actual time, are displayed. Furthermore, the user can filter the daily route by time, e.g., to view peak times more precisely. This display is available for both historical routes and for optimized routes, so that the schedules can be compared geographically.
2. *Gantt Chart* A Gantt chart is a traditional project management tool that graphically depicts the time sequence of activities in the form of bars on a timeline (cf. [40]). In our tool, the user can visualize the chronological sequence of a schedule in a Gantt chart, which also groups different tasks that belong to the same transport vehicle. For each vehicle, both its shift time and the operations carried out are displayed. The Gantt chart can be adjusted interactively so that delays are highlighted in color or transports are divided into their individual sections. In addition, further details such as the workload, delays and shift violations are displayed for each vehicle. This enables the user to quickly identify which vehicles fulfill certain objectives and which do not. Furthermore, we are able to visualize whether a vehicle is a (known) Covid-19 transport. Figure 10 shows an example of a Gantt chart for the vehicles on the left hand side with two delays of 15 and 32 min, respectively.

#### Challenges and outcome

The developed decision-support system cannot be used yet at the control center. Nevertheless, there are many insights from the optimal solution that are helpful in practice. Furthermore, practical recommendations on how to determine optimized plans can be derived. As an example recommendation that resulted from the mathematical optimization studies that is already used in practice, we mention the incorporation of so-called *dummy transports* for the return trips from dialysis appointments. These dummy transports can be created when information about a transport is partially available, This is, in the case of dialysis, the origin and destination of the return trip that are known from the outward trip. In contrast, the exact time is not known beforehand and can only be estimated. Based on this estimation, a dummy transport is created that is also integrated in the optimization process. This is described in more detail in [39]. In addition, a more general question from the control center was studied, namely whether it is advisable to plan with more dummy transports in order to be prepared for additional future requests that typically arise. However, as the patient transport system is under strong pressure, considerably more dummy transports easily lead to delays in the whole system, so that the recommendation was to include them carefully.

## Discussion

The previously introduced decision-support tools were all developed with and demonstrated to experts in the respective domains and received positive feedback. We have been able to visualize our mathematical solutions in a compact and visually appealing way. Solutions can be interactively explored with respect to different goals in mind. However, a sustainable integration into the planners’ processes and software systems is a complex undertaking. The most problematic concerns in this respect are (data) security, data provision and accuracy, integration, and scalability issues.

*Security* The presented decision support tools were implemented as web-based services with a client-server architecture which has many advantages. First of all, modern web-UI libraries already offer a variety of feature-rich, interactive components that can be customized to meet specific needs. Used examples are the leaflet-react library [41], which allows to create interactive maps, the DHTMLX Gantt library [42] and the Ant-Design (Pro) library [43] which provides—among others—a variety of charting and selection components.

The developed architecture also allows for an integration of remote procedure calls to optimization routines that may be stored on different servers and are potentially written in different programming languages. When releasing a decision support tool to a planner, it is advantageous to host a server by an admin who installs and maintains the required frameworks, while the planner only needs a modern web browser to access it.

However, in the field of emergency rescue and patient transport, the data that necessarily has to be exchanged between the server and the browser, contains highly sensitive data, which, for example, contains the coordinates and timestamps of emergencies and the corresponding actions. Therefore, such a transfer, if desired, must be protected by state-of-the-art encryption and web-socket technology. In our studies, we did not take the risk of data privacy breaches and installed these services only locally.

**Fig. 11:**
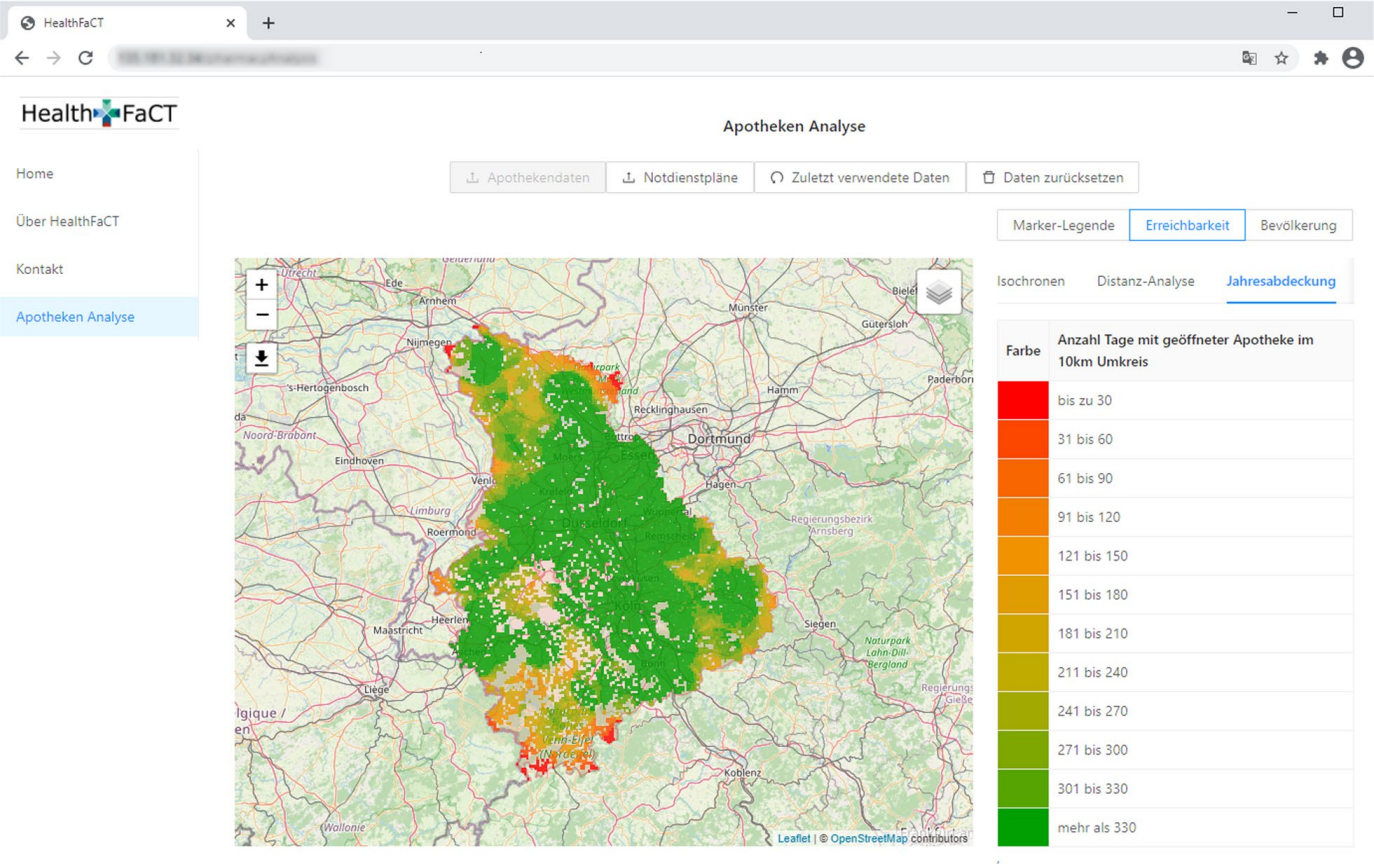
Exemplary screenshot of the web-based decision support platform

Pharmacy data is less sensitive and most of it is anyhow publicly available. Therefore, the tool for analyzing out-of-hours plans is already hosted and can be accessed after a login (see Fig. 11 for a screenshot).

*Data Provision and Accuracy* The software uses a variety of data for the analyses where some data is stored statically in the back end and other data has to be dynamically uploaded by users. The static data includes in particular the street graphs of the pilot regions (obtained via [18]) as well as data on municipalities and districts within the considered region (e.g. geographic representations as shape-files). These do not change very often, but must be maintained by the service provider. The dynamic data sets vary depending on the application. In the current implementation, it is the specification of an optimized solution. The quality of community data for the use in street networks is subject to scientific research (cf. [44, 45]) and is of course debatable. For strategic questions such as the erection of a rescue station, a temporal snapshot may be misleading when a major road is temporarily closed or some erroneous additions are not yet corrected. A commercially prepared street data set often has a finer distinction of street classes, which is relevant for the estimation of speed profiles. However, all data sets are prone to decay and are never completely reliable, neither for the historic situation nor for future situations. Nevertheless, our personal communication to experts from the planning of emergency infrastructure revealed that they do trust in open street map data often more than in commercially acquired data sets.

The preparation of historical emergency data sets for the use in optimization and analysis algorithms is even more complex. The timestamps and coordinates have to be recorded semiautomatically by pressing certain buttons. Due to human or technical errors, a large quantity of records may still either be incomplete, wrong or missing completely. For a one-time analysis and consult, it is often sufficient to focus on the reliable part of the records. But, the determination of a reliable data set requires a significant manual effort. In a productive use of such a tool, this must be replaced by some learned heuristic rules.

*Integration* In a regularly arising strategic or tactical planning question, it may already be a show stopper that planners have to provide updated data sets which are compatible with a software tool. Beyond that, in our experience, the usage of third-party tools is generally accepted as long as they are intuitive and rewarding with respect to the planning quality. The use of third-party tools in an operational context is much more problematic. The most important challenge is that data must be synchronized between the individual systems. Therefore, direct communication interfaces via APIs or a common database access are mandatory (while in strategic tools file-based interfaces are mostly sufficient). This communication usually must work both ways. In the fourth case study "Minimization of waiting time in patient transport logistics", this means that a new patient transport request must be available to the planning algorithm and its scheduling decision must be send to the control center software. Communication must work in real-time and the optimizer also has small time windows to return a decision. In the referenced case study, the usage of a third-party optimization software would not even have been legal unless officially certified.

*Scalability* Another frequent issue in the transition from a demonstrator to a productive tool is the variation of input sizes. For the case studies described above, it is reasonable to restrict e.g. the street network to the planning regions. Their sizes and hence the isochrone computation may vary vastly between different administrative districts, which must be tested and treated in advance when considering new regions. In general, also the running times of most optimization algorithms increase with larger problem sizes. This could be the number of emergency stations or pharmacies to consider or the number of transportation requests to handle. In a productive tool, limitations or estimated running times must be approximated beforehand in order to avoid user frustration.

When multiple users access the tool simultaneously, the software must be designed in such a way that parallel access does not lead to inconsistent data situations. One way to solve this, is a pure read-only mode of the back end. This is however a very limiting scenario. Alternatively, session cookies need to be stored in order to distinguish the respective data sets. If the use case requires multiple people to make write changes to the same data, a database can be integrated that manages the access orders. Depending on the planners’ hierarchy, it usually also makes sense to define different roles with varying permissions within a team. As an example, there may be multiple planners that can analyze a location or shift plan, but only the senior planner can overwrite the currently executed plan.

## Conclusions and outlook

The tools developed here support experts in ambulatory healthcare by suggesting best possible decisions for several of its main pillars, namely for pharmacies, emergency doctors, vaccination stations and for patient transport in different regions of Germany. The tools could naturally be generalized to other regions. Beyond the presented case studies, further healthcare planning problems could be optimized with individual optimization models and algorithms. On the one hande, the presented visualization tools are versatile enough to be reused in a wide variety of domains and specific planning questions. On the other hand, individual adaptations to the targeted planners and their use cases are required to give them maximum benefit, namely to transparently communicate the value of the optimized solutions and trade-offs.

In summary, it leads to a versatile toolbox that has already received positive feedback from practitioners. Additional application opportunities arise due to the fast developments in digitalization of the healthcare sector. Joining data- as well as model-driven tools for an integrated digital rescue chain poses many interesting mathematical and algorithmic research topics.

Nevertheless, some challenges arise when it comes to using the tool in practical operation. For long-term planning as described here, the tool can be and has already been used for the derivation of optimized plans. For operative planning, e.g. in patient transport or for emergency doctors, however, mainly security issues currently still prevent its usage in real operative planning. There is a reasonable chance that these hurdles can be overcome in collaboration with the application experts and dispatchers, so that the benefits of mathematical optimization can be made available in the future.

## Data Availability

The datasets generated and/or analysed during the current study are not publicly available due to confidentially agreements but are available from the corresponding author on reasonable request.

## Footnotes

1 A class of problems in complexity theory, that can intuitively be considered as problems which can most likely not be solved in polynomial time. For a formal definition we refer to [21].

## Declarations

### Concerning human involvement

There were no human participants or human data involved in the study.
